# CLCA1 suppresses colorectal cancer aggressiveness via inhibition of the Wnt/beta-catenin signaling pathway

**DOI:** 10.1186/s12964-017-0192-z

**Published:** 2017-10-03

**Authors:** Xiaofen Li, Wangxiong Hu, Jiaojiao Zhou, Yanqin Huang, Jiaping Peng, Ying Yuan, Jiekai Yu, Shu Zheng

**Affiliations:** 1 Cancer Institute (Key Laboratory of Cancer Prevention and Intervention, China National Ministry of Education, China), the Second Affiliated Hospital, Zhejiang University School of Medicine, Hangzhou, Zhejiang China; 2 Department of Abdominal Oncology, West China Hospital, Sichuan University, Chengdu, Sichuan China; 3 Department of Medical Oncology, the Second Affiliated Hospital, Zhejiang University School of Medicine, Hangzhou, Zhejiang China

**Keywords:** Chloride channel accessory 1, Colorectal cancer, Tumor suppressor, Early detection

## Abstract

**Background:**

Chloride channel accessory 1 (CLCA1) belongs to the calcium-sensitive chloride conductance protein family, which is mainly expressed in the colon, small intestine and appendix. This study was conducted to investigate the functions and mechanisms of CLCA1 in colorectal cancer (CRC).

**Methods:**

The CLCA1 protein expression level in CRC patients was evaluated by enzyme-linked immunosorbent assay (ELISA), immunohistochemistry (IHC), and western blotting analysis. Using CRISPR/Cas9 technology, CLCA1-upregulated (CLCA1-ACT) and CLCA1-knockout cells (CLCA1-KO), as well as their respective negative controls (CLCA1-ACT-NC and CLCA1-KO-NC), were constructed from the SW620 cell line. Cell growth and metastatic ability were assessed both in vitro and in vivo. The association of CLCA1 with epithelial-mesenchymal transition (EMT) and other signaling pathways was determined by western blotting assays.

**Results:**

The expression level of CLCA1 in CRC tissues was significantly decreased compared with that in adjacent normal tissue (*P*< 0.05). Meanwhile, the serum concentration of CLCA1 in CRC patients was also significantly lower when compared with that of healthy controls (1.48 ± 1.06 ng/mL vs 1.06 ± 0.73 ng/mL, *P* = 0.0018). In addition, CLCA1 serum concentration and mRNA expression level in CRC tissues were inversely correlated with CRC metastasis and tumor stage. Upregulated CLCA1 suppressed CRC growth and metastasis in vitro and in vivo, whereas inhibition of CLCA1 led to the opposite results. Increased expression levels of CLCA1 could repress Wnt signaling and the EMT process in CRC cells.

**Conclusions:**

Our findings suggest that increased expression levels of CLCA1 can suppress CRC aggressiveness. CLCA1 functions as a tumor suppressor possibly via inhibition of the Wnt/beta-catenin signaling pathway and the EMT process.

**Electronic supplementary material:**

The online version of this article (dio: 10.1186/s12964-017-0192-z) contains supplementary material, which is available to authorized users.

## Background

The human chloride channel accessory proteins (CLCA) are a family of secreted self-cleaving proteins that activate calcium-dependent chloride currents [1, 2]. The CLCA family comprises three members, named CLCA1, 2 and 4 [3, 4]. *CLCA3* is a truncated pseudogene and does not encode a protein [5, 6]. All members of this protein family share a high degree of homology in protein size, sequence, and predicted structure but differ significantly in tissue distribution and biological functions [3, 4]. CLCA1, the first reported human CLCA family member, is mainly expressed in the large and small intestine, especially in crypt cells, and can be secreted into the blood [7]. The second CLCA isoform, CLCA2, is highly expressed in the trachea and mammary glands [8], while CLCA4 is mostly expressed in neural tissue (note that CLCA4 was misidentified as CLCA2 in the study conducted by Agnel M, et al.) [9]. Upon their discovery in 1998, the functions of human CLCA proteins were mostly thought to be associated with calcium-dependent chloride conductance [7–9]. As research continues, more interesting and valuable functions of the CLCA family have been identified, such as involvement in mucus secretion and tumor metastasis and regulation of the cell cycle, apoptosis, and blood pressure [3, 10–15].

The important role of certain ion channels in tumor progression is well acknowledged [16]. These channels influence cell volume, intracellular pH, the concentration of signaling molecules, and the activity of protein kinases and phosphatases by regulating ion currents [17]. For example, voltage-dependent anion channel 1 (VDAC1) is overexpressed in many cancer types. In addition, downregulation of VDAC1 expression inhibits tumor development [18]. It has been reported that potassium channel subfamily K member 9 (KCNK9) is frequently overexpressed in breast cancer. In addition, in vitro experiments indicate that the overexpression of KCNK9 promotes tumor formation and is associated with tumor resistance to hypoxia and serum deprivation [19]. There is a growing body of evidence showing that CLCA proteins, which act on calcium-dependent chloride channels and facilitate chloride conductance, have a close relationship with tumor progression [15, 20–22]. For instance, studies have validated that the loss of CLCA2 expression is closely associated with tumorigenicity and the metastasis of breast cancer [15, 20, 21]. Secreted CLCA1 has been demonstrated to be a direct modulator of TMEM16A, a member of the calcium-dependent chloride channel family [23] [24],. Recently, several studies have suggested that CLCA1 is downregulated in tumors, and its repression predicts poor prognosis [25–27]. In our previous proteogenomic study using mass spectrometry and gene microarray, we determined that CLCA1 protein and gene expression levels are dramatically reduced in CRC tissue compared with adjacent normal mucosa, suggesting that CLCA1 is a potential biomarker of CRC [28]. However, the biological functions and molecular mechanisms of CLCA1 in colorectal cancer (CRC) remain to be elucidated.

By using a cohort of CRC blood and tissue samples collected from the Second Affiliated Hospital of Zhejiang University School of Medicine between 2015 and 2016, we found that the expression level of CLCA1 in CRC patient tissues/serum was markedly decreased compared with that in adjacent normal tissues/healthy controls. Moreover, CLCA1 serum concentration and the *CLCA1* mRNA expression level were inversely correlated with CRC metastasis and tumor stage. To further investigate the functions and mechanisms of CLCA1 in CRC, we used CRISPR/Cas9 technology to construct CLCA1-upregulated cells (CLCA1-ACT) and CLCA1-knockout cells (CLCA1-KO) in the SW620 cell line. In vitro and in vivo experiments revealed that the increased expression level of CLCA1 suppressed CRC proliferation and metastasis, whereas inhibition of CLCA1 caused the opposite effects. An increased expression of CLCA1 was able to repress Wnt signaling and the EMT process in CRC cells. These results suggest that CLCA1 may function as a tumor suppressor in CRC by inhibiting the Wnt/beta-catenin signaling pathway and EMT process. As far as we know, this is the first study investigating the role of CLCA1 in CRC in vivo.

## Methods

### Sample collection and patient characteristics

The serum samples used for enzyme-linked immunosorbent assay (ELISA) were collected from 76 healthy volunteers and 100 CRC patients prior to treatment, who were admitted to the Second Affiliated Hospital, Zhejiang University School of Medicine between 2015 and 2016. All serum samples were stored in a refrigerator at −80 °C. For immunohistochemistry (IHC) analysis, paired CRC and adjacent normal tissues were surgically collected from 32 patients, fixed in 10% buffered formalin and embedded in paraffin. For western blotting analysis, 19 pairs of CRC and adjacent normal tissues were surgically obtained and frozen at −80 °C. Patient clinical characteristics such as age, gender and TNM stage are listed in the Additional file 1. Written informed consent was obtained from each patient. The Ethics Committee of the Second Affiliated Hospital, Zhejiang University School of Medicine approved this study.

### Enzyme-linked immunosorbent assay (ELISA)

Serum levels of CLCA1 in CRC patients and healthy controls were measured using a commercially available CLCA1 sandwich ELISA kit according to the manufacturer’s protocol (USCN Life Science, SEG669Hu). All samples and standards were detected in duplicate.

### IHC staining and semi-quantitative analysis

IHC staining was performed with the avidin–biotin–peroxidase complex method. Briefly, paraffin-embedded blocks were sectioned at ~5-μm thickness. Slides were baked at 60 °C overnight, deparaffinized with xylene and rehydrated in a graded ethanol series. After the antigen retrieval process, endogenous peroxidase inactivity and preincubation in 10% normal goat serum, the sections were incubated with the anti-CLCA1 antibody (1:1000 dilution, Abcam, ab180851) at room temperature for 2 h and then with the peroxidase-polymer labeled secondary antibody. Then, peroxidase activity was demonstrated with diaminobenzidine. Finally, sections were counterstained with hematoxylin.

Two independent pathologists evaluated the staining expression based on both the intensity and distribution of positive cells. Staining intensity was graded as follows: 0, absent; 1, weak staining; 2, moderate staining; and 3, strong staining. Staining distribution was determined by the percentage of positive cells (0, <5% positive cells; 1, 5–25% positive cells; 2, 26–50% positive cells; and 3, >50% positive cells). The two scores were summed and divided by 2 to obtain the final score, which was categorized as negative (−) for scores <2 or positive (+) for scores ≥2.

### Western blotting analysis

Protein extracted from fresh-frozen tissues was loaded and separated by 10% SDS-polyacrylamide gel electrophoresis (SDS-PAGE). Then, the proteins were transferred onto polyvinylidene fluoride (PVDF) membranes by electroblotting and incubated with primary antibodies.

Immunoreactive bands were detected by chemiluminescence using corresponding horseradish peroxidase (HRP)-conjugated secondary antibodies and enhanced chemiluminescence (ECL) detection reagents. Gray intensity analysis of the western blot images was conducted by ImageJ software. Then, relative protein abundance was determined.

Primary antibodies used for western blot include anti-CLCA1 (1:1000 dilution, Abcam, ab180851), anti-GAPDH (1:1000, Cell Signaling Technology, #5174), anti-beta-catenin (1:1000, Cell Signaling Technology, #8480), anti-E-cadherin (1:1000, Cell Signaling Technology, #3195), anti-N-cadherin (1:1000, Cell Signaling Technology, #13116), anti-vimentin (1:1000, Cell Signaling Technology, #5741), anti-slug (1:1000, Cell Signaling Technology, #9585), anti-snail (1:1000, Cell Signaling Technology, #3879), anti-p53 (1:1000, Cell Signaling Technology, #2527), anti-Akt (1:1000, Cell Signaling Technology, #4691), anti-Ras (1:1000, Cell Signaling Technology, #14429), anti-NF-kappa B (1:1000, Cell Signaling Technology, #8242) and anti-histone H3 (1:2000, Cell Signaling Technology, #4499).

### The cancer genome atlas (TCGA) database analysis

All normal (*n* = 51) and CRC (*n* = 625) level 3 *CLCA1* gene expression datasets were obtained from the TCGA database (October 2015). To obtain a high-confidence result, we only considered HiSeq samples for messenger RNA (mRNA) (RNASeqV2). RSEM-normalized data for *CLCA1* was log_2_-transformed for better visualization. The boxplot and statistical analysis were performed in the R programming language (×64, version 3.0.2).

### Quantitative reverse transcription polymerase chain reaction (qRT-PCR)

Total RNA was extracted from fresh-frozen tissues or CRC cells. The Takara PrimeScriptTM RT Master Mix kit (Takara, RR036Q) was used for reverse transcription. The SYBR Premix Ex Taq II kit (Takara, RR820A) and Applied Biosystems 7500 Fast Real-Time PCR System were applied for real time PCR analysis. Experiments were carried out in triplicate, and GAPDH was used as the loading control. The forward primer sequence of *CLCA1* was CGTCAAATACTCCCCATCGT (5′ to 3′), and the reverse primer sequence was GCTGATGTTCTGGTTGCTGA (5′ to 3′). The forward primer sequence of GAPDH was ATCCCATCACCATCTTCCAG (5′ to 3′), and the reverse primer sequence was TGAGTCCTTCCACGATACCA (5′ to 3′). The ΔΔCt method was applied to evaluate the mRNA relative expression level.

### Cell culture and plasmid transfection

SW620 cells, which were bought from the American Type Culture Collection, were cultured in Leibovitz L-15 medium at 37 °C in 5% CO_2_. Culture medium was supplemented with 10% fetal bovine serum (FBS, Gibco, 10,100,139), 100 U/mL penicillin and 100 mg/mL streptomycin. CLCA1 CRISPR/Cas9 (the Clustered Regularly Interspaced Short Palindromic Repeats and CRISPR-associated protein system) activation plasmid (sc-402,998-ACT) and CLCA1 CRISPR/Cas9 knockout plasmid (double nickase plasmid, sc-402,998-NIC) were bought from Santa Cruz Biotechnology and transfected into SW620 cells according to the manufacturer’s protocols. To obtain stable transfectants of the activation plasmid (CLCA1-ACT), blasticidin, hygromycin and zeocin were used to select successfully transfected SW620 cells. In addition, to obtain stable transfectants of the knockout plasmid (CLCA1-KO), puromycin was used to select cells. Meanwhile, non-targeting plasmids were transfected in the same way for the negative controls as for CLCA1-ACT and CLCA1-KO, i.e., CLCA1-ACT-NC and CLCA1-KO-NC, respectively.

### Cell proliferation analysis

Cell Counting Kit-8 (CCK-8, KeyGen) was applied to evaluate cell proliferation. Experiments were performed according to the manufacturer’s protocol. Briefly, 1 × 10^4^ cells were seeded in a 96-well plate containing 100 μL of completed culture medium per well and incubated in a 37 °C incubator. Culture medium was used as a blank control. Cell proliferation was evaluated every day for approximately 1 week after plating. CCK-8 solution (10 μL) was added to each well, and then, the plate was incubated with cells in the 37 °C incubator for 2 h. An optimal density (OD) value of 450 nm was used to measure cell proliferation. The mean and SD were calculated from 3 independent assays.

### Cell colony formation assay

Cell colony formation experiments were performed to reflect anchorage-independent cell growth. Approximately 1000 cells were seeded in a 6-well plate containing complete culture medium and incubated in a 37 °C incubator. Colonies consisting of more than 50 cells after 2 weeks were counted.

### Cell migration and invasion assay

A transwell chamber with an 8-μm-pore filter membrane (Corning Inc.) was used to evaluate cell migration. Cells (2 × 10^5^) in serum-free medium were seeded into the upper chamber, while conditioned medium with 20% FBS was added to the lower chamber. The chambers were incubated for 48 h in a 37 °C incubator. Non-migrated cells in the upper chamber were removed with cotton swabs. Migrated cells on the underside of the filter membrane were fixed in 4% (*v*/v) paraformaldehyde and stained with crystal violet. The Matrigel-coated 8-μm-pore transwell chamber (Corning Inc.) was used to evaluate cell invasion. The procedures of the cell invasion assay were identical to the cell migration assay. The migrated/invaded cells were counted by light microscopy, and the mean cell number of five random visual fields at a magnification of 200× was recorded. The assays were carried out in triplicate.

### Construction of animal models

Animal experiments were conducted according to the Animal Study Guidelines of Zhejiang University. Five-week-old, female nude mice (BALB/C) were used for the animal study. To construct the subcutaneous tumor xenograft mouse model, 5 × 10^6^ tumor cells were injected subcutaneously at the costal margin. The size of the xenograft tumor was measured every 3 days. The mice were killed 6 weeks later, and the subcutaneous xenograft tumors were dissected and weighed. In the meantime, to construct a liver metastasis mouse model, 2.5 × 10^6^ tumor cells were injected into the tail vein. After 8 weeks, the mice were killed, and the number of liver metastases was enumerated and fixed in 10% formalin.

### Statistical analysis

SPSS Statistics 20.0 (IBM, Armonk, NY, USA) was used to conduct statistical analysis. Statistical tests were two-sided, and *P*< 0.05 was considered statistically significant. Chi-square tests were performed to compare qualitative data; two-tailed Student’s *t*-tests were used to compare quantitative data.

## Results

### Decreased expression of CLCA1 in CRC

**Fig. 1:**
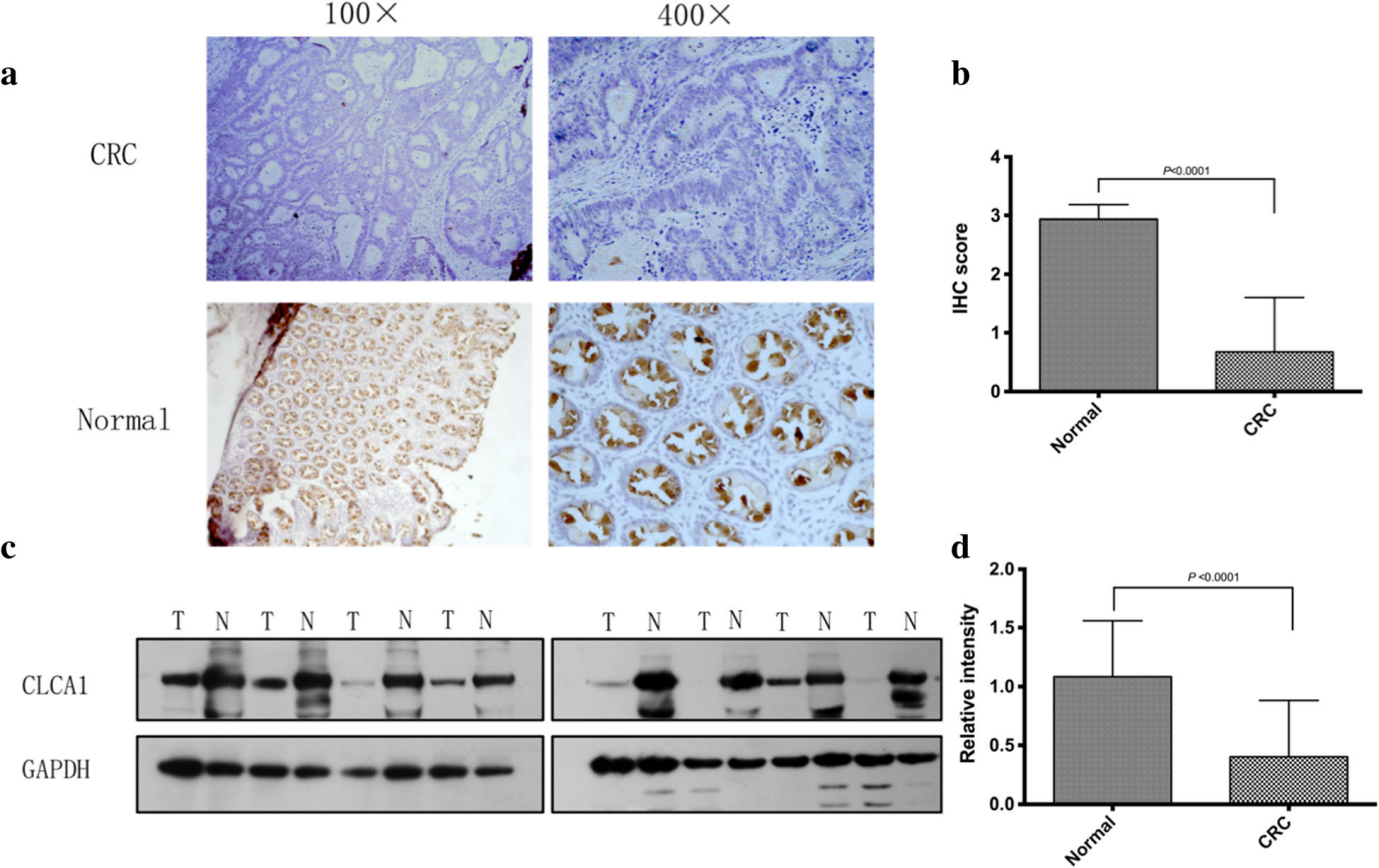
Decreased expression level of CLCA1 in CRC tissues. **a** Representative images of IHC staining of CLCA1 in paraffin-embedded CRC tissues and matched adjacent normal mucosa. **b** IHC staining scores in the CRC group and adjacent normal tissues (*P* < 0.0001, paired *t*-test). **c** Representative bands from western blotting of matched fresh-frozen CRC and adjacent normal mucosa. **d** Relative CLCA1 protein intensity in the CRC group and adjacent normal tissues (*P* < 0.0001, paired *t*-test). Abbreviations: IHC, immunohistochemistry; GAPDH, glyceraldehyde-3-phosphate dehydrogenase

**Fig. 2:**
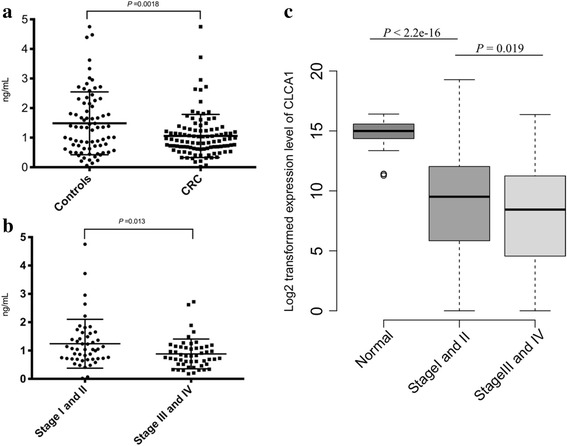
CLCA1 serum concentration and *CLCA1* mRNA expression level are inversely correlated with CRC metastasis. **a** ELISA results showed that the CLCA1 serum expression level was significantly lower in CRC patients than in healthy controls (1.48 ± 1.06 ng/mL vs 1.06 ± 0.73 ng/mL, *P* = 0.0018, *t*-test). **b** Compared to that in patients with early stage disease (TNM stage I and II), the CLCA1 serum expression level was markedly reduced in patients with local or distant metastasis (TNM stage III and IV) (1.24 ± 0.86 ng/mL vs 0.88 ± 0.53 ng/mL, *P* = 0.013, *t*-test). **c** *CLCA1* mRNA level in CRC samples from TCGA database. Abbreviations**:** ELISA, enzyme-linked immunosorbent assay; TCGA, the Cancer Genome Atlas

To evaluate the expression level of CLCA1 in CRC tissue, we performed IHC and western blotting analysis in pairs of CRC and adjacent normal tissues. Thirty-two pairs of paraffin-embedded CRC and adjacent normal mucosa were used for the IHC analysis. The results showed that the IHC staining score in the normal group was significantly higher than that in the CRC group (2.94 ± 0.25 vs 0.67 ± 0.93, *P* < 0.0001, paired *t*-test, Fig. 1a and b). In addition, the CLCA1-positive rate in the normal group was 100% (32/32), which was in sharp contrast with the mere 12.5% (4/32) in the CRC group (*P*< 0.0001, chi-square test). Western blotting analysis of the 19 pairs of fresh-frozen CRC and adjacent normal mucosa confirmed the IHC findings, revealing markedly decreased expression levels of CLCA1 in CRC tissue compared to that in the adjacent normal mucosa (*P*< 0.0001, paired *t*-test, Fig. 1c and d). We next analyzed the CLCA1 serum expression level in 100 CRC patients and 76 healthy controls by ELISA and found that the CLCA1 serum expression level was significantly lower in CRC patients than in healthy controls (1.48 ± 1.06 ng/mL vs 1.06 ± 0.73 ng/mL, *P* = 0.0018, *t*-test, Fig. 2a). In addition, compared to that in patients with early-stage CRC (TNM stage I and II), the CLCA1 serum expression level was significantly reduced in patients with local or distant metastasis (TNM stage III and IV) (1.24 ± 0.86 ng/mL vs 0.88 ± 0.53 ng/mL, *P* = 0.013, *t*-test, Fig. 2b).

To further validate the *CLCA1* mRNA expression level in CRC, we analyzed 51 normal intestinal mucosa and 625 CRC samples from TCGA database. Similarly, the *CLCA1* mRNA expression level was significantly lower in CRC tissue than in adjacent normal tissue (*P*< 2.2e-16, *t*-test, Fig. 2c). Further analysis showed that the expression level of *CLCA1* was decreased greatly in stage III/IV compared with stage I/II (*P* = 0.019, *t*-test, Fig. 2c), which was in accordance with the results of the ELISA, suggesting that CLCA1 is involved in CRC metastasis.

These results demonstrated a decreased expression level of CLCA1 in CRC tissues and patient serum. In addition, the CLCA1 serum concentration and *CLCA1* mRNA expression level were inversely correlated with CRC metastasis and tumor stage.

### Increased expression of CLCA1 suppresses CRC cell growth and metastasis in vitro

**Fig. 3:**
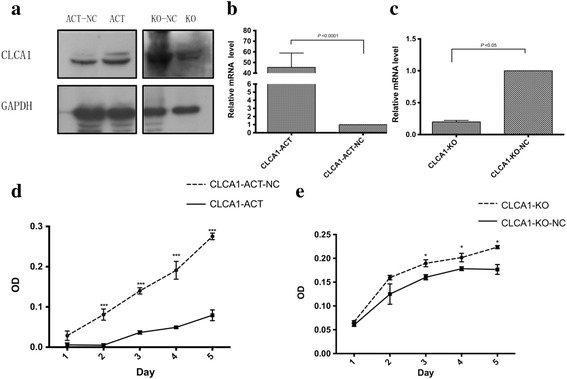
Increased expression level of CLCA1 suppresses CRC cell growth in vitro. **a** Western blotting analysis of CLCA1 in the SW620 cell line transfected with CLCA1 CRISPR/Cas9-activation and -knockout plasmids. **b** qRT-PCR analysis of *CLCA1* in the SW620 cell line transfected with CLCA1 CRISPR/Cas9-activation plasmids. **c** qRT-PCR analysis of *CLCA1* in SW620 cell line transfected with CLCA1 CRISPR/Cas9-knockout plasmids. **d** Growth curves of the CLCA1-ACT and CLCA1-ACT-NC group. **e** Growth curves of the CLCA1-KO and CLCA1-KO-NC group. Abbreviations: qRT-PCR, quantitative reverse transcription polymerase chain reaction; CRISPR/Cas9, the Clustered Regularly Interspaced Short Palindromic Repeats and CRISPR-associated protein system; CLCA1-ACT, CLCA1-upregulated cells; CLCA1-ACT-NC, negative control of CLCA1-upregulated cells; CLCA1-KO, CLCA1-knockout cells; CLCA1-KO-NC, negative control of CLCA1-knockout cells. *P < 0.05, *t*-test; ***P < 0.001, *t*-test

To clarify the role of CLCA1 action in CRC tumorigenesis and metastasis, we constructed stable CLCA1-upregulated (CLCA1-ACT) and CLCA1-knockout cells (CLCA1-KO) in the SW620 cell line, as well as the respective negative control cells (CLCA1-ACT-NC and CLCA1-KO-NC), using CRISPR/Cas9 plasmids (Fig. 3a, b and c).

CCK-8 analysis was performed to evaluate cell proliferation. The results showed that from day 2 to day 5, the mean absorbance of the CLCA1-ACT group was dramatically lower than the CLCA1-ACT-NC group (*P*< 0.05, *t*-test, Fig. 3d). In contrast, on days 3 to 5, the mean absorbance of the CLCA1-KO group was significantly higher than that of the CLCA1-KO-NC group (*P*< 0.05, *t*-test, Fig. 3e). The growth curves of CLCA1-ACT and CLCA1-KO cells suggested that the increased expression of CLCA1 was able to suppress SW620 cell proliferation, whereas inhibition of CLCA1 was able to promote SW620 cell proliferation.

**Fig. 4:**
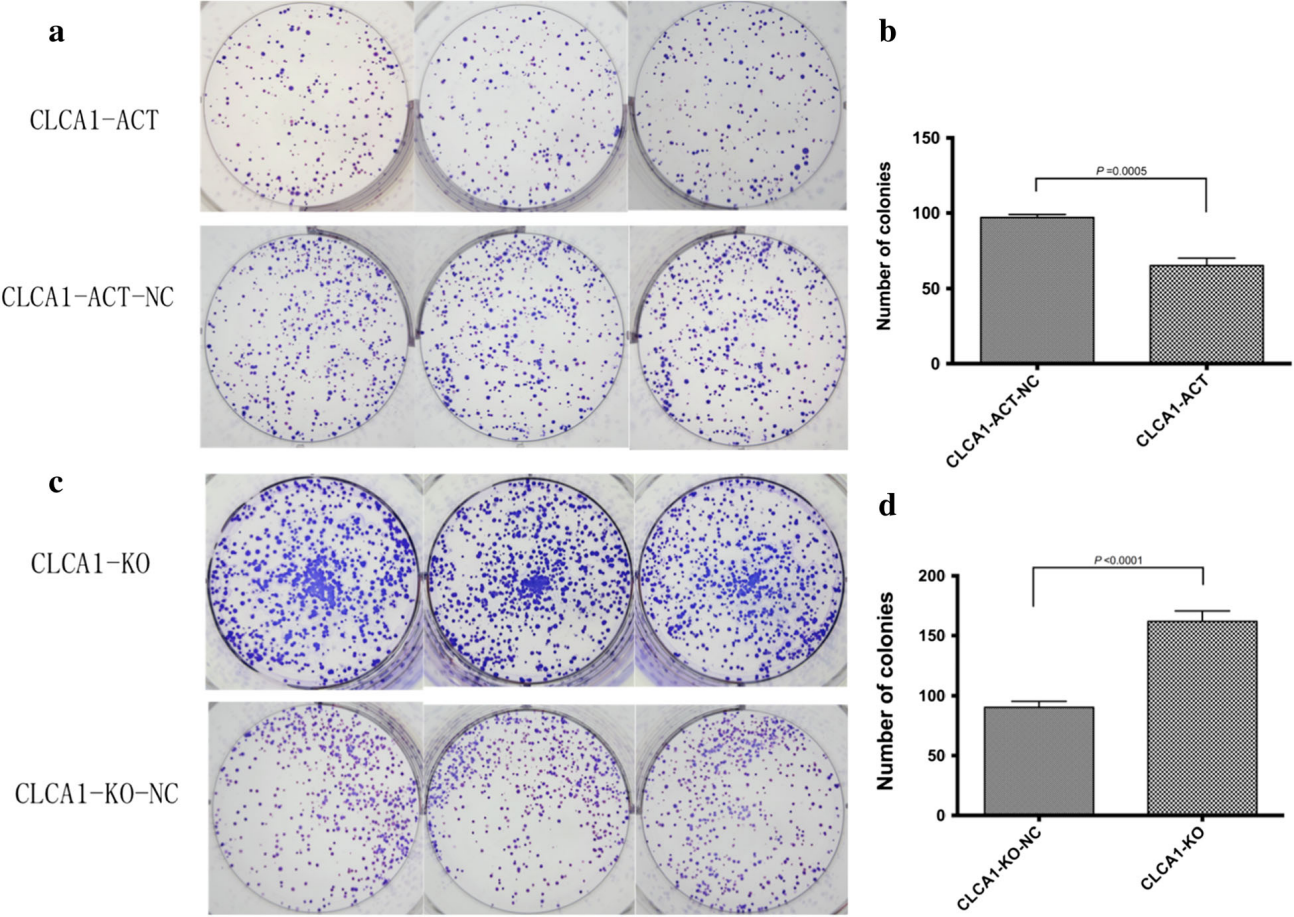
Increased expression level of CLCA1 suppresses CRC cell colony formation ability in vitro. **a** Images of the colony formation assay with CLCA1-ACT and CLCA1-ACT-NC cells. **b** The mean number of colonies in CLCA1-ACT and CLCA1-ACT-NC cells (*P* < 0.0001 *t*-test). **c** Images of the colony formation assay with CLCA1-KO and CLCA1-KO-NC cells. **d** Mean number of colonies in the CLCA1-KO and CLCA1-KO-NC cells (*P* < 0.0001, *t*-test). Abbreviations: CLCA1-ACT, CLCA1-upregulated cells; CLCA1-ACT-NC, negative control of CLCA1-upregulated cells; CLCA1-KO, CLCA1-knockout cells; CLCA1-KO-NC, negative control of CLCA1-knockout cells

Consistently, the results of the colony formation assay showed that the upregulation of CLCA1 retarded anchorage-independent cell growth, while the inhibition of CLCA1 promoted this ability (Fig. 4a and c).

**Fig. 5:**
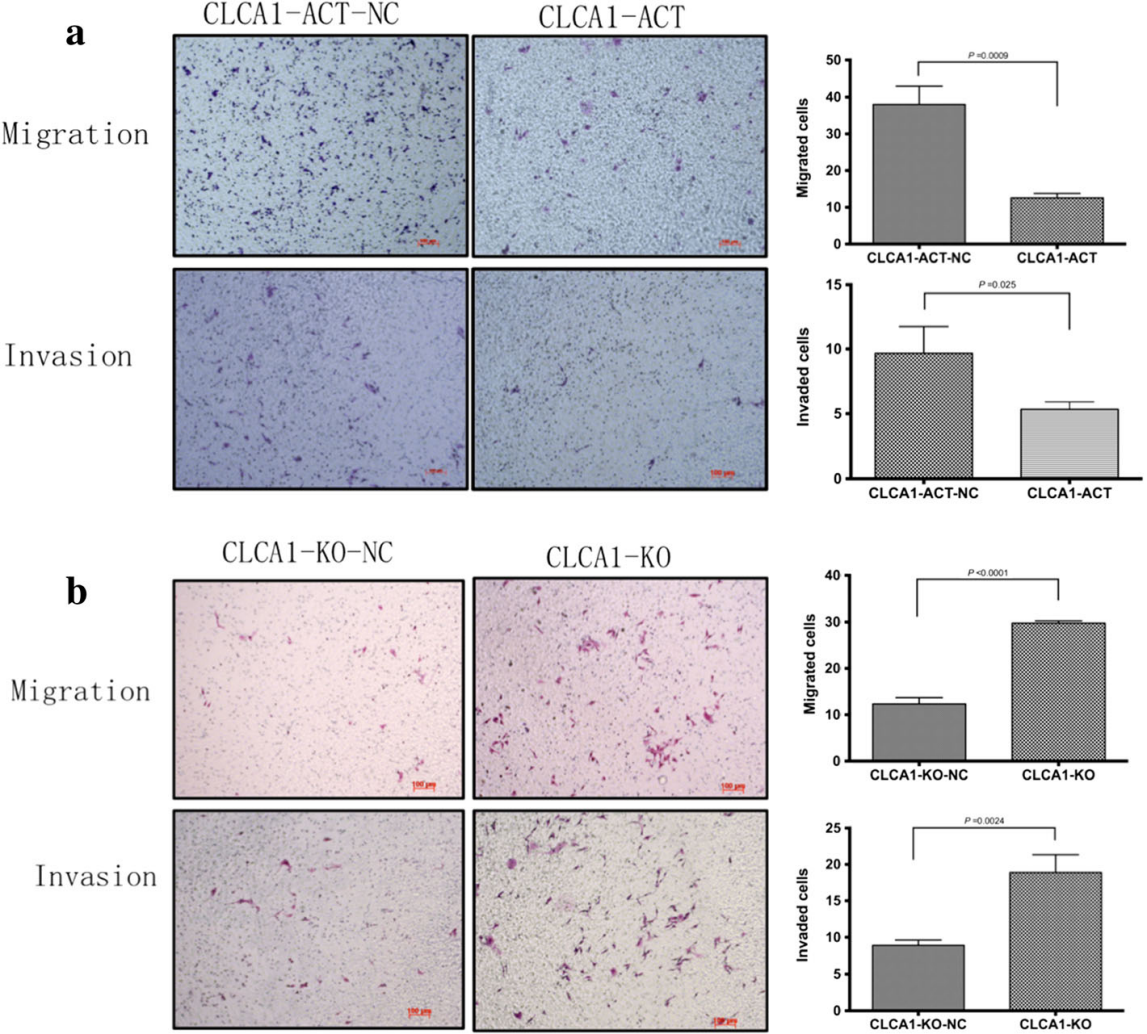
Increased expression level of CLCA1 reduces CRC cell migration and invasion in vitro. **a** Cell migration and invasion assays in CLCA1-ACT and CLCA1-ACT-NC cells (left panel, representative pictures of transwell chambers; right panel, average counts of five random microscopic fields at a magnification of 200×). **b** Cell migration and invasion assays in CLCA1-KO and CLCA1-KO-NC cells (left panel, representative pictures of transwell chambers; right panel, average counts of five random microscopic fields at a magnification of 200×)

To further evaluate the impacts of CLCA1 on CRC metastasis, a transwell chamber with an 8-μm-pore filter membrane was used to measure cell migration. Meanwhile, Matrigel-coated 8-μm-pore transwell chambers were used to evaluate cell invasion ability. Migrating/invading cells on the underside of the filter membrane were stained and counted. The results revealed that both the migration and invasion properties of CLCA1-ACT cells were significantly reduced compared with that of CLCA1-ACT-NC cells (Fig. 5a). In contrast, both the migration and invasion properties of CLCA1-KO cells were much more than that of the negative control cells (Fig. 5b). These results indicated that the increased expression level of CLCA1 was able to reduce cell migration and invasion, while inhibition of CLCA1 was able to enhance cell migration and invasion.

Collectively, our data suggested that the increased expression level of CLCA1 probably suppressed CRC cell growth and metastasis, whereas inhibition of CLCA1 led to the opposite results.

### Increased expression of CLCA1 inhibits CRC proliferation and metastasis in vivo

**Fig. 6:**
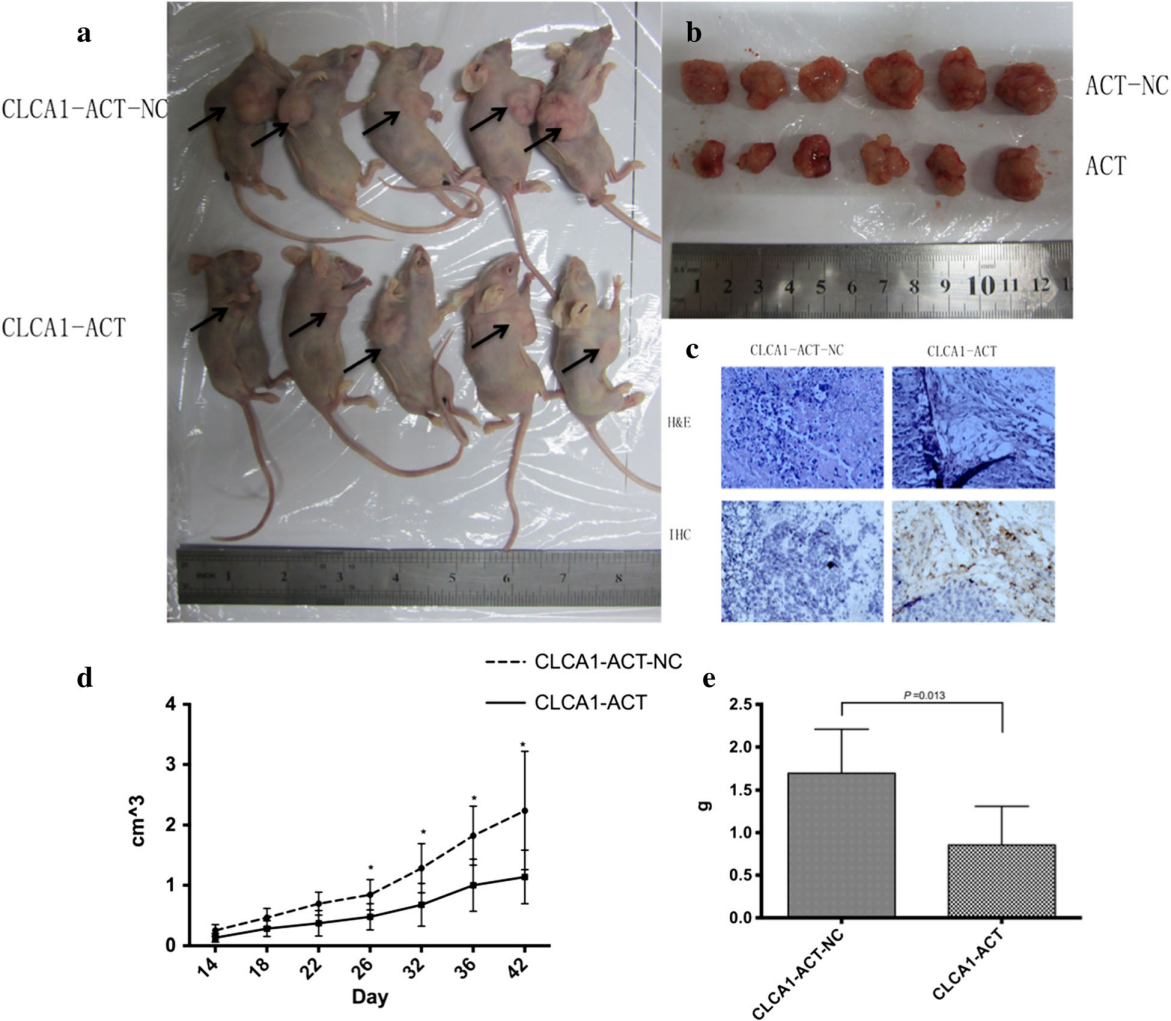
Increased expression level of CLCA1 inhibits CRC proliferation and metastasis in vivo. **a** Subcutaneous xenograft mouse models (arrows show subcutaneous tumors). **b** Dissected subcutaneous xenograft tumors. **c** Representative H & E staining images and CLCA1 IHC staining images of xenograft tumors. **d** Subcutaneous xenograft tumor growth curves of the CLCA1-ACT and CLCA1-ACT-NC group. **e** Average tumor weight of the CLCA1-ACT and CLCA1-ACT-NC group. Abbreviations: H & E, Hematoxylin and eosin; IHC, immunohistochemistry

To further confirm the impact of CLCA1 on tumor growth and metastasis in vivo, tumor xenograft mouse models were constructed. CLCA1-ACT cells and negative controls (5 × 10^6^ cells per mouse) were injected subcutaneously into mice. Tumor volume was measured every 3 days, and thereby, the tumor growth curve was plotted. The mice were killed 6 weeks after the injection, and the subcutaneous xenograft tumors were dissected and weighed (Fig. 6a, b, and c). Figure 6d and e show the xenograft tumor growth curves and average tumor weight of the CLCA1-ACT and CLCA1-ACT-NC group, suggesting that the increased expression of CLCA1 inhibited CRC proliferation in vivo.

**Fig. 7:**
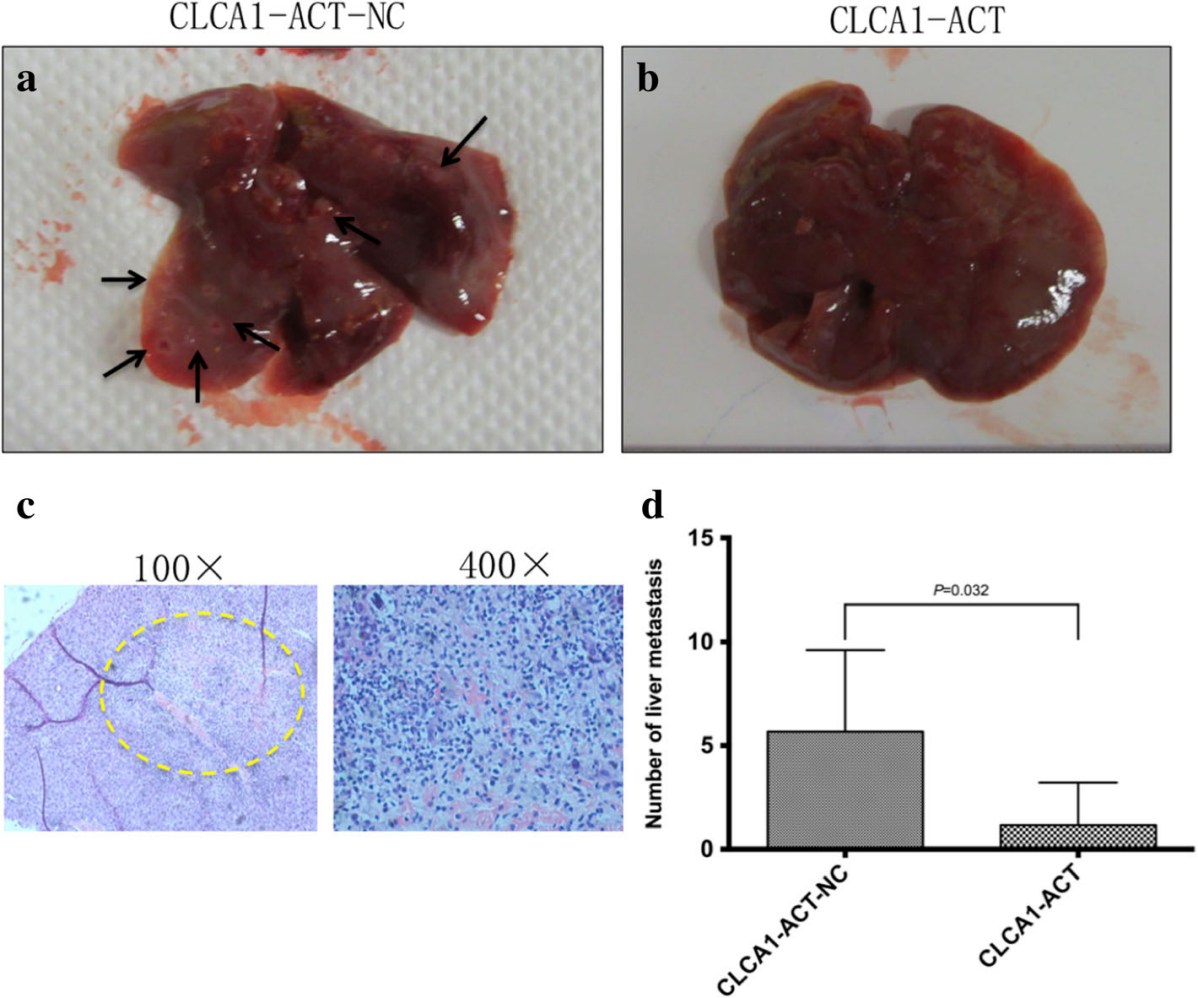
Increased expression level of CLCA1 inhibits CRC metastasis in vivo. **a** Representative liver with metastatic tumors (arrows) from the CLCA1-ACT-NC group. **b** Representative liver without visible metastatic tumors from the CLCA1-ACT group. **c** H & E staining image of liver metastasis in the CLCA1-ACT group. **d** Average number of visible liver metastatic nodules

Additionally, liver metastasis mouse models were constructed by injecting CLCA1-ACT and CLCA1-ACT-NC cells into the tail veins (2.5 × 10^6^ cells per mouse). Eight weeks after the injection, the mice were killed, and liver metastases were enumerated and fixed in 10% formalin. We found that the average number of visible liver metastatic nodules in the CLCA1-ACT group was markedly smaller than that of the CLCA1-ACT-NC group (Fig. 7).

Taken together, these results revealed that the increased expression of CLCA1 had a critical role in suppressing CRC growth and metastasis in vivo.

### The mechanism of CLCA1 inhibition of CRC aggressiveness

#### CLCA1 upregulation inhibits the epithelial-mesenchymal transition (EMT)

**Fig. 8:**
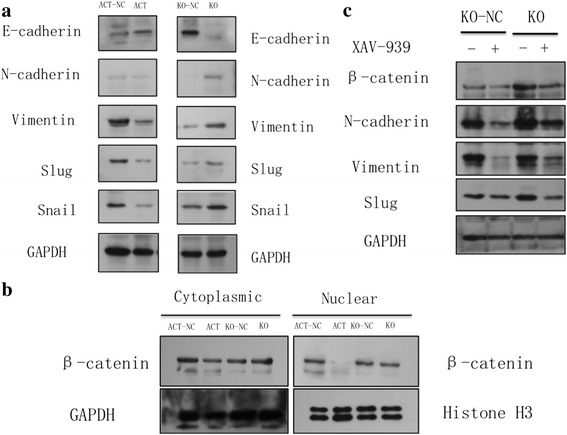
CLCA1 expression level upregulation inhibits EMT and Wnt/beta-catenin signaling. **a** Western blotting analysis showed that the increased expression level of CLCA1 increased the E-cadherin expression level but repressed N-cadherin, vimentin, slug, and snail expression levels. CLCA1 expression level downregulation caused the reverse effects. **b** Beta-catenin nuclear translocation was decreased after CLCA1 expression level was upregulated and increased after CLCA1 was knocked down. **c** The expression levels of beta-catenin and mesenchymal markers were downregulated in CLCA1-KO cells after treatment with XAV939. Abbreviations**:** EMT, epithelial-mesenchymal transition

To investigate the functional mechanism of CLCA1 in CRC, we performed western blotting analysis of the proteins related to EMT. The results revealed that the increased expression level of CLCA1 increased the E-cadherin expression level but repressed N-cadherin, vimentin, slug, and snail expression levels (Fig. 8a). Conversely, CLCA1 downregulation decreased the E-cadherin expression level but enhanced N-cadherin, vimentin, slug, and Snail expression levels (Fig. 8a). It is well known that E-cadherin is an epithelial marker, while N-cadherin, vimentin, slug and Snail are mesenchymal markers. These results indicated that CLCA1 upregulation possibly inhibits EMT, which is a process where polarized epithelial cells are converted into non-polarized mesenchymal cells; during this process, migration and invasion abilities are improved.

#### CLCA1 upregulation inhibits the Wnt/beta-catenin signaling pathway

Markers of several main signaling pathways such as the p53, Wnt, PI3K, NF-kappa B and Ras/MAPK pathways were detected by western blotting analysis (see Additional file 2). The results showed that beta-catenin, which is a marker of the Wnt pathway, was downregulated in the CLCA1-ACT transfectants but upregulated in the CLCA1-KO transfectants. In addition, beta-catenin nuclear translocation, which is a marker of Wnt signaling activation, was decreased after CLCA1 was activated and increased after CLCA1 was knocked down (Fig. 8b). These results strongly suggested that the increased expression of CLCA1 might inhibit the Wnt/beta-catenin signaling pathway.

**Fig. 9:**
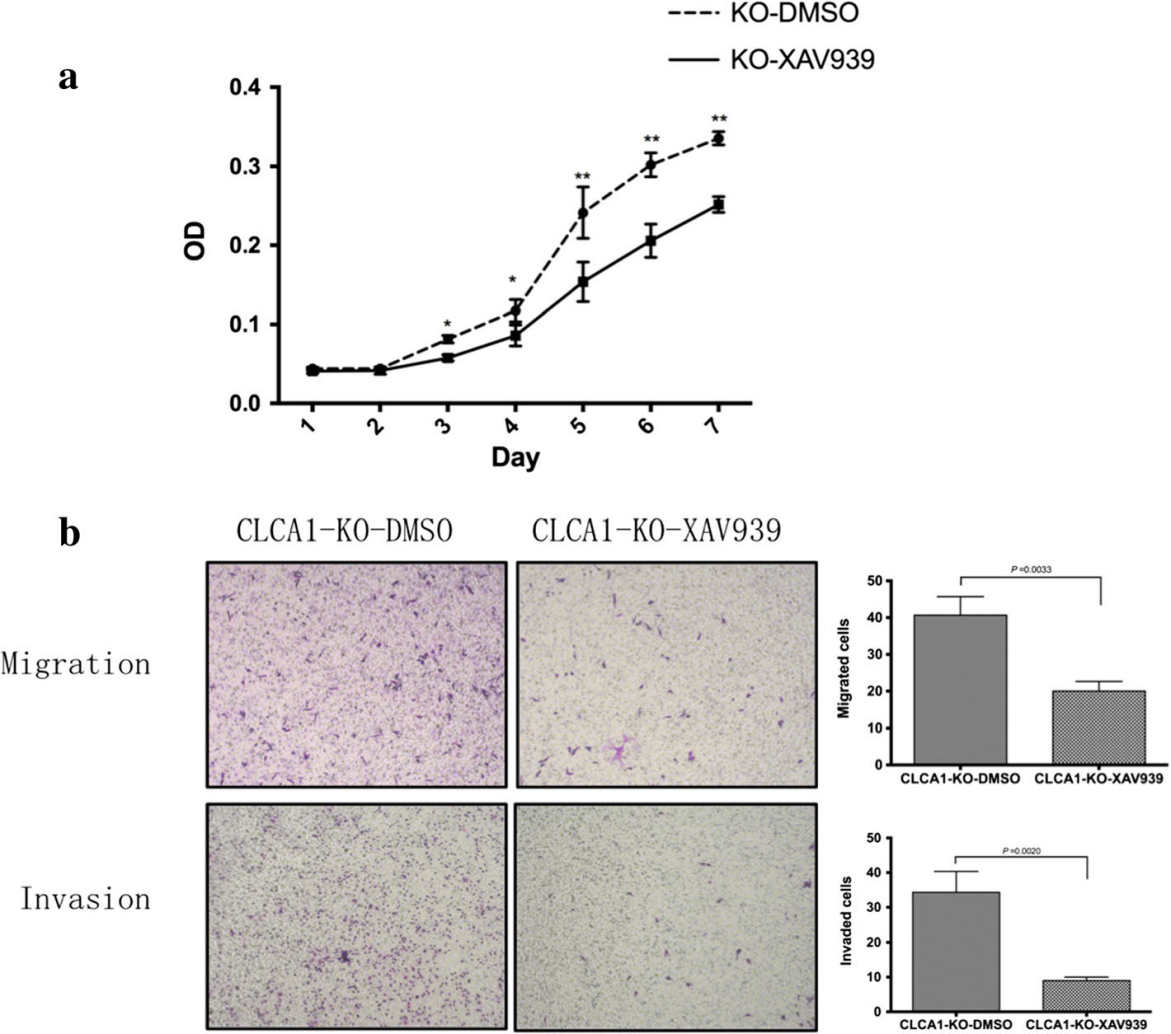
Promotion of CRC cell aggressiveness was abrogated when CLCA1-KO cells were co-treated with XAV939. **a** The promotion of CRC cell growth was abrogated when CLCA1-KO cells were co-treated with XAV939. **b** The enhancement CRC cell migration and invasion was abrogated when CLCA1-KO cells were co-treated with XAV939 (left panel, representative pictures of transwell chambers; right panel, average counts of five random microscopic fields at a magnification of 200×). *P < 0.05, *t*-test; **P < 0.01, *t*-test

To confirm this speculation, we treated CLCA1-KO cells with the Wnt signaling pathway specific inhibitor, XAV939. Dimethylsulfoxide (DMSO) was used as a negative control. It was observed that, compared to that in CLCA1-KO cells treated with DMSO, the expression levels of beta-catenin and proteins associated with the epithelial-mesenchymal transition (EMT) were downregulated in CLCA1-KO cells treated with XAV939 (Fig. 8c). We next assessed cell proliferation and metastasis again. As described before, inhibition of CLCA1 promoted CRC growth and metastasis, but we observed that these effects could be abrogated when cells were co-treated with XAV939 (Fig. 9a and b). These phenomena also confirmed the association of CLCA1 with the Wnt pathway from the reverse side.

## Discussion

CRC is the third most commonly diagnosed malignancy worldwide and is the fourth leading cause of cancer-related deaths [29]. The five-year survival rates of CRC patients in diagnosed at different stages vary dramatically, from more than 70% in early stage-diagnosed patients to less than 10% in those diagnosed at advanced stages [30]. More than half of patients have regional or distant metastasis at the time of diagnosis [28]. Thus, early detection could significantly improve CRC survival. Until now, there has not been a highly sensitive or specific biomarker for CRC diagnosis or monitoring.

In this study, we evaluated the association between CLCA1 and CRC development and investigated the biological functions and mechanisms of CLCA1 in CRC. Our results demonstrated that the expression level of CLCA1 in CRC tissues was markedly decreased compared with that in adjacent normal mucosa. Compared with the healthy controls, CLCA1 serum concentration in CRC patients was dramatically reduced. In addition, both CLCA1 serum concentration and *CLCA1* mRNA expression level were inversely correlated with CRC metastasis and tumor stage. The in vitro and in vivo experiments suggested that the increased expression of CLCA1 suppressed CRC cell proliferation and metastasis, while the inhibition of CLCA1 caused the reverse effects. Western blotting analysis indicated that the Wnt signaling pathway was activated and EMT was induced when CLCA1 was inhibited. Furthermore, these effects were abrogated when cells were co-treated with the Wnt signaling specific inhibitor, XAV939. In conclusion, we believe that increased expression levels of CLCA1 can suppress CRC aggressiveness. In addition, CLCA1 functions as a tumor suppressor possibly by downregulating the Wnt/beta-catenin signaling pathway and EMT.

Several studies have suggested that CLCA family proteins play vital roles in tumor progression [13, 15, 20–22, 25–27, 31, 32]. Early in 1999, Gruber A.D. and his colleagues [20] determined that the expression of CLCA2 was frequently lost in breast cancer and that the re-expression of CLCA2 repressed tumor metastasis in vitro and in vivo. It has been concluded that CLCA2 may act as a tumor suppressor in breast cancer. This was verified in later studies conducted by Walia V. et al. [15, 21]. Moreover, their results also indicated that CLCA2 is a p53-inducible growth inhibitor [21] and that the loss of CLCA2 promotes EMT in breast cancer [15]. Similarly, it has been reported that CLCA4 is downregulated and promotes EMT in breast cancer, which indicates a tumor-suppression function for CLCA4 [22]. In 2001, Bustin S.A. et al. [25] preliminarily observed that the expression levels of the *CLCA1* and *CLCA2* genes were significantly downregulated in CRC. The excellent work of Yang B. and his team indicated that low expression of CLCA1 predicts CRC recurrence and lower survival [27]. CLCA1 contributes to promoting spontaneous differentiation and reducing CRC cell proliferation in vitro [26]. Similar conclusions have been obtained in murine and porcine CLCA isoforms [33–35].

The conclusions of our study are in accordance with previous reports [25–27]. Furthermore, for the first time, we have investigated the functions of CLCA1 in vivo using stably transfected cells and confirmed the association of CLCA1 with EMT and the Wnt signaling pathway. It is well-known that a great majority of CRC patients carry mutations in the adenomatous polyposis coli (*APC*) or beta-catenin (*CTNNB1*) gene and that both genes are involved in the Wnt/beta-catenin signaling pathway [36–40]. The aberrant activation of Wnt signaling induces the cytoplasmic accumulation and nuclear translocation of beta-catenin protein [40, 41], which is a vital mechanism involved in cancer cell proliferation and metastasis. As reported, aberrant Wnt signaling can trigger the EMT process, which also plays a crucial role in cancer metastasis [42]. During the EMT process, epithelial cells convert to mesenchymal cells, losing cell-cell adhesion and cell polarity and acquiring migratory and invasive properties [43, 44]. In our study, the increased expression of CLCA1 reduced the beta-catenin expression level and repressed EMT, which probably explains the tumor-inhibitory activity of CLCA1.

The outstanding work of Sala-Rabanal M. and her colleagues demonstrates that CLCA1 can stabilize TMEM16A on the cell surface and prevent its internalization, thus activating chloride currents [23, 24]. In addition, several studies have indicated that TMEM16A is overexpressed in certain cancers and closely associated with tumor progression [45, 46]. Therefore, we hypothesize that the tumor-suppressor function of CLCA1 might be related to TMEM16A stabilization and thus reduce its tumor promotion ability, which needs further investigation.

We do acknowledge that some limitations exist in our study. For instance, we only included one CRC cell line for our laboratory study, which may result in cell line bias. In addition, the sample size of our clinical research was limited by the difficulties of sample collection and preservation. We are trying to collect more samples to validate our conclusions. Furthermore, the precise mechanism of how CLCA1 interacts with beta-catenin or other proteins participating in the Wnt signaling pathway remains to be determined. Related experiments, such as expression profile sequencing and co-immunoprecipitation, are in progress.

In summary, we demonstrated that CLCA1 is downregulated in CRC tissues and patient serum, suggesting that CLCA1 may serve as a novel biomarker for the early diagnosis of CRC. Both in vitro and in vivo experiments revealed that the increased expression level of CLCA1 was able to suppress CRC aggressiveness, which is associated with inhibition of the Wnt signaling pathway and EMT. These findings indicate that CLCA1 may function as a tumor suppressor, but future efforts are needed to elucidate the role of CLCA1 in the Wnt/beta-catenin signaling network.

## Additional files

Additional file 1: Patients’ clinical characteristics in IHC, western blotting analysis and ELISA. **Table S1.** listed patients’ clinical characteristics in IHC experiment and IHC staining results. **Table S2.** and **S3.** descripted patients’ clinical characteristics in western blotting analysis and ELISA, respectively. (DOC 62 kb)

Additional file 2: Western blotting images of markers of p53, Wnt, PI3K, NF-kappa B and Ras/MAPK signaling pathway. The results showed that beta-catenin, which is a marker of the Wnt pathway, was downregulated in the CLCA1-ACT transfectants but upregulated in the CLCA1-KO transfectants. (PNG 118 kb)

## Abbreviations

CCK-8: Cell Counting Kit-8
CLCA1: Chloride channel accessory 1
CLCA1-ACT: CLCA1-upregulated cells
CLCA1-ACT-NC: Negative controls of CLCA1-ACT
CLCA1-KO: CLCA1-knockout cells
CLCA1-KO-NC: Negative controls of CLCA1-KO
CRC: Colorectal cancer
CRISPR/Cas9: Clustered regularly interspaced short palindromic repeats and CRISPR-associated protein system
DMSO: Dimethylsulfoxide; APC, adenomatous polyposis coli
ECL: Enhanced chemiluminescence
ELISA: Enzyme-linked immunosorbent assay
EMT: Epithelial-mesenchymal transition
GAPDH: Glyceraldehyde-3-phosphate dehydrogenase
HRP: Horseradish peroxidase
IHC: Immunohistochemistry
KCNK9: Potassium channel subfamily K member 9
MET: Mesenchymal-epithelial transition
MRNA: Messenger RNA
OD: Optimal density
PVDF: Polyvinylidene fluoride
qRT-PCR: Quantitative reverse transcription polymerase chain reaction
SDS-PAGE: SDS-polyacrylamide gel electrophoresis
TCGA: The Cancer Genome Atlas
VDAC1: Voltage-dependent anion channel 1
