# Supplementary material for: CLCA1 suppresses colorectal cancer aggressiveness via inhibition of the Wnt/beta-catenin signaling pathway
Source: Cell Commun Signal. 2017 Oct 3;15:38. doi: 10.1186/s12964-017-0192-z (PMC5627483; doi:10.1186/s12964-017-0192-z)
Supplement: Supplementary file 1 — Patients’ clinical characteristics in IHC, western blotting analysis and ELISA. Table S1. listed patients’ clinical characteristics in IHC experiment and IHC staining results. Table S2. and S3. descripted patients’ clinical characteristics in western blotting analysis and ELISA, respectively. (DOC 62 kb) [file 12964_2017_192_MOESM1_ESM.doc]

Table S1 Patients’ characteristics and CLCA1 IHC staining results

| Variables | N (%) | Rate of IHC score≥1 (%) | *P* |
| --- | --- | --- | --- |
| *Age (years old)* |  |  |  |
| ≤65 | 17(53.1) | 7/17(41.2) | 0.946 |
| >65 | 15(46.9) | 6/15(40) |  |
| *Gender* |  |  |  |
| Male | 16(50.0) | 5/16(31.3) | 0.280 |
| Female | 16(50.0) | 8/16(50) |  |
| *Primary tumor site* |  |  |  |
| Left-sided | 22(68.7) | 6/22(27.3) | 0.023 |
| Right-sided | 10(31.3) | 7/10(70.0) |  |
| *Primary tumor stage* |  |  |  |
| T1/T2 | 6(18.8) | 1/6(16.7) | 0.185 |
| T3/T4 | 26(81.2) | 12/26(46.2) |  |
| *Lymph node involvement* |  |  |  |
| Negative | 18(56.3) | 7/18(38.9) | 0.821 |
| Positive | 14(43.7) | 6/14(42.9) |  |
| *Distant metastasis* |  |  |  |
| No | 29(90.6) | 12/29(41.4) | 0.787 |
| Yes | 3(9.4) | 1/3(33.3) |  |
| *TNM stage* |  |  |  |
| Stage I/II | 16(50.0) | 6/16(37.5) | 0.719 |
| Stage III/IV | 16(50.0) | 7/16(43.8) |  |

Table S2 Patients’ characteristics in western blotting analysis

| Variables | N (%) |
| --- | --- |
| *Age (years old)* |  |
| ≤65 | 10(52.6) |
| >65 | 9(47.4) |
| *Gender* |  |
| Male | 9(47.4) |
| Female | 10(52.6) |
| *Primary tumor site* |  |
| Left-sided | 13(68.4) |
| Right-sided | 6(31.6) |
| *TNM stage* |  |
| Stage I/II | 12(63.2) |
| Stage III/IV | 7(36.8) |

Table S3 Patients’ clinical features in ELISA

| Variables | N (%) | CLCA1 serum concentration (ng/mL) | *P* |
| --- | --- | --- | --- |
| *Age (years old)* |  |  |  |
| ≤65 | 57(57) | 0.99±0.62 | 0.27 |
| >65 | 43(43) | 1.15±0.85 |  |
| *Gender* |  |  |  |
| Male | 59(59) | 1.08±0.70 | 0.71 |
| Female | 41(41) | 1.02±0.78 |  |
| *TNM stage* |  |  |  |
| Stage I/II | 50(50) | 0.88±0.53 | 0.013 |
| Stage III/IV | 50(50) | 1.24±0.86 |  |
