# Supplementary figures and images for: CLCA1 suppresses colorectal cancer aggressiveness via inhibition of the Wnt/beta-catenin signaling pathway
Source: Cell Commun Signal. 2017 Oct 3;15:38. doi: 10.1186/s12964-017-0192-z (PMC5627483; doi:10.1186/s12964-017-0192-z)

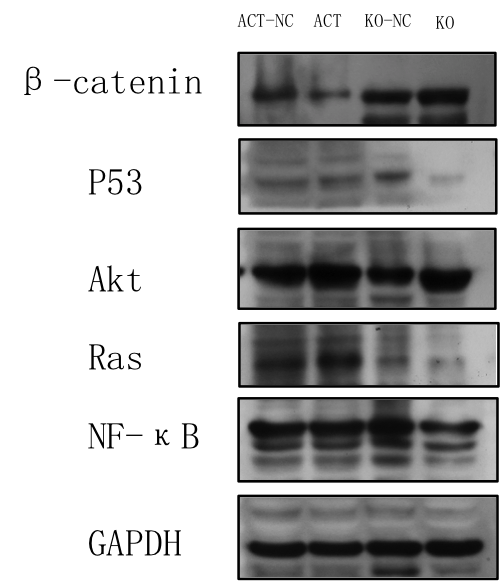

Supplement: Supplementary file 2 — Western blotting images of markers of p53, Wnt, PI3K, NF-kappa B and Ras/MAPK signaling pathway. The results showed that beta-catenin, which is a marker of the Wnt pathway, was downregulated in the CLCA1-ACT transfectants but upregulated in the CLCA1-KO transfectants. (PNG 118 kb) [file 12964_2017_192_MOESM2_ESM.png]
